# Novel Immune-Related Gene Signature for Risk Stratification and Prognosis of Survival in Lower-Grade Glioma

**DOI:** 10.3389/fgene.2020.00363

**Published:** 2020-04-15

**Authors:** Mingwei Zhang, Xuezhen Wang, Xiaoping Chen, Qiuyu Zhang, Jinsheng Hong

**Affiliations:** ^1^Department of Radiation Oncology, The First Affiliated Hospital of Fujian Medical University, Fuzhou, China; ^2^Institute of Immunotherapy, Fujian Medical University, Fuzhou, China; ^3^Key Laboratory of Radiation Biology (Fujian Medical University), Fujian Province University, Fuzhou, China; ^4^Fujian Key Laboratory of Individualized Active Immunotherapy, Fuzhou, China; ^5^Fujian Medical University Union Hospital, Fuzhou, China; ^6^Department of Statistics, College of Mathematics and Informatics & FJKLMAA, Fujian Normal University, Fuzhou, China

**Keywords:** lower grade glioma, The Cancer Genome Atlas, Chinese Glioma Genome Atlas, immune-related signature, prognosis

## Abstract

**Objective:**

Despite several clinicopathological factors being integrated as prognostic biomarkers, the individual variants and risk stratification have not been fully elucidated in lower grade glioma (LGG). With the prevalence of gene expression profiling in LGG, and based on the critical role of the immune microenvironment, the aim of our study was to develop an immune-related signature for risk stratification and prognosis prediction in LGG.

**Methods:**

RNA-sequencing data from The Cancer Genome Atlas (TCGA), Genome Tissue Expression (GTEx), and Chinese Glioma Genome Atlas (CGGA) were used. Immune-related genes were obtained from the Immunology Database and Analysis Portal (ImmPort). Univariate, multivariate cox regression, and Lasso regression were employed to identify differentially expressed immune-related genes (DEGs) and establish the signature. A nomogram was constructed, and its performance was evaluated by Harrell’s concordance index (C-index), receiver operating characteristic (ROC), and calibration curves. Relationships between the risk score and tumor-infiltrating immune cell abundances were evaluated using CIBERSORTx and TIMER.

**Results:**

Noted, 277 immune-related DEGs were identified. Consecutively, 6 immune genes (*CANX*, *HSPA1B*, *KLRC2*, *PSMC6*, *RFXAP*, and *TAP1*) were identified as risk signature and Kaplan–Meier curve, ROC curve, and risk plot verified its performance in TCGA and CGGA datasets. Univariate and multivariate Cox regression indicated that the risk group was an independent predictor in primary LGG. The prognostic signature showed fair accuracy for 3- and 5-year overall survival in both internal (TCGA) and external (CGGA) validation cohorts. However, predictive performance was poor in the recurrent LGG cohort. The CIBERSORTx algorithm revealed that naïve CD4^+^ T cells were significant higher in low-risk group. Conversely, the infiltration levels of M1-type macrophages, M2-type macrophages, and CD8^+^T cells were significant higher in high-risk group in both TCGA and CGGA cohorts.

**Conclusion:**

The present study constructed a robust six immune-related gene signature and established a prognostic nomogram effective in risk stratification and prediction of overall survival in primary LGG.

## Introduction

Lower-grade gliomas (LGG) constitute the prevalent primary malignances of the central nervous system, demonstrating great intrinsic heterogeneity in terms of their biological behavior (Ostrom et al., 2013; Zeng et al., 2018). So far, maximum surgical resection combined with postoperative radiotherapy and chemotherapy is the standard treatment for LGG. Despite numerous efforts to improve the clinical outcome, more than half of the LGG cases evolve and progress to therapy-resistant high-grade aggressive glioma over time (Claus et al., 2015). Thus, it is imperative to identify novel prognostic factors for LGG. Several biomarkers, including the isocitrate dehydrogenase (IDH) mutation, co-deletion of chromosome arms 1p and 19q (1p/19q codeletion), and O-6-methylguanine-DNA methyltransferase (MGMT) methylation have been integrated to the 2016 WHO classification, to illustrate the histological features and guide the therapeutic strategy (Hartmann et al., 2010; Wick et al., 2013; Hainfellner et al., 2014; Louis et al., 2016). However, these widely utilized biomarkers do not fully elucidate the individual variants and properly address risk stratification in LGG. Thus, it would only be reasonable to attempt to integrate various methods, including gene expression profiles that have gathered enormous attention, to further improve stratification of LGG.

The immune microenvironment has been identified as playing a critical role in tumor biology (Hanahan and Weinberg, 2011), and recently, numerous promising preclinical and clinical immunotherapeutic treatments, including immune-checkpoint inhibitors, active or passive immunotherapy, and gene therapy, have been achieved in malignant gliomas (Mahmoodzadeh Hosseini et al., 2015; Xu et al., 2015; Reznik et al., 2018; Simonelli et al., 2018; Vismara et al., 2019), further establishing the vital role of immunotherapy in the management of gliomas. Hence, the molecular profiles of the immune components within the tumor microenvironments represent tremendous value in serving as prognostic biomarkers. Recently, several studies have proposed immune gene expression-based signatures for risk stratification and for predicting clinical outcomes in breast, gastric, thyroid, and ovarian cancers (Ascierto et al., 2012; Kim et al., 2018; Shen et al., 2019; Yang et al., 2019). In terms of the prognostic value of an immune-related risk signature in glioma, Cheng et al. (2016) revealed that not only did the immune-related risk signature had prognostic significance in the stratified patients for glioblastoma, but moreover the immune status and local immune response could be illustrated by the risk signature. However, implementation of an immune gene expression-based signature has not been fully elucidated in LGG.

In a previous study, Li and Meng (2019) identified an immune-related long non-coding RNA (lncRNA) signature based on 529 low-grade glioma cases. It was found that the 8-lncRNAs model could serve as an independent predictor in low-grade glioma, not enrolling cases of grade III glioma. However, the predictive accuracy of the lncRNA-based model needed to be enhanced and the external validation was warranted. Furthermore, the correlation between the immune-related model and immune cell phenotypes was not illustrated. To our knowledge, the latest version of Cell type Identification By Estimating Relative Subsets Of RNA Transcripts (CIBERSORTx) has been investigated as a highly sensitive and specific algorithm set to reveal the immune landscape of 22 human immune cell compositions in solid tumors (Newman et al., 2019) and thus might provide new insights into potential therapeutic candidates for the management of LGG.

In the present study, a large cohort of patients with primary LGG from The Cancer Genome Atlas (TCGA) database and normal control cases from the Genome Tissue Expression (GTEx) database were employed to screen differentially expressed immune-related genes (IRGs). After construction of the risk signature based on the immune related genes, patients with primary LGG with gene sequencing data from the Chinese Glioma Genome Atlas (CGGA) database were adopted as the external validation. In addition, the CIBERSORTx and Tumor Immune Estimation Resource (TIMER) algorithm were utilized to clarify the correlation between the risk signature and the abundances of the infiltrative immune cells in primary LGG samples.

## Materials and Methods

### Acquisition of LGG Expression Profiles From TCGA Datasets

The RNA-seq data (level 3) and clinical information of LGG samples were collected from UCSC Xena^1^. Expression of genes analyzed in normal tissues was collected using the Genome Tissue Expression (GTEx) (Consortium, 2015; Gentles et al., 2015) tool. Normalized gene expression was measured as fragments per kilobase of transcript per million mapped reads (FPKM) and log2-based transformation. Then, the “sva” package of R software was utilized for the normalization of RNA expression profiles and to remove the batch effects. Principal component analysis (PCA) was used for detecting batch effects from the GTEx and TCGA datasets.

### Acquisition of Immune-Related Genes

A comprehensive list of IRGs was downloaded from the Immunology Database and Analysis Portal (ImmPort) database^2^. The list comprised a total of 2,498 IRGs, covering 17 immune categories (Bhattacharya et al., 2014).

### Inclusive and Exclusive Criteria of Enrolled Patients for the Construction of Risk Signature

The inclusive criteria of patients with LGG for model construction were as follows: (1) only patients with primary glioma were enrolled, (2) pathologic types of WHO II or III grade, (3) complete clinicopathological parameters, (4) only samples with RNA-sequencing data, (5) overall survival (OS) as the primary endpoint, (6) minimum follow-up of 90 days. The exclusive criteria included (1) patients with recurrent LGG, (2) pathologic type was glioblastoma, (3) incomplete survival status and clinical information.

### Establishment of the Immune-Related Risk Signature

Using the “survival” package in R, we employed univariate Cox regression on IRGs and OS of primary LGG in the TCGA database to identify survival-associated IRGs. Next, using the “glmnet” package in R, the least absolute shrinkage and selection operator (Lasso) regression model was selected to minimize the over-fitting and identify the most significant survival-associated IRGs in primary LGG. After testing for collinearity, stepwise multivariate Cox regression analysis was performed to establish the IRG-derived risk signature in primary LGG. The following formula based on a combination of Cox coefficient and gene expression was used to calculate the risk score (Lossos et al., 2004; Chen et al., 2007; Hu et al., 2019):

$$M\,o\,d\,e\,l:R\,i\,s\,k\,s\,c\,o\,r\,e=\sum_{i=1}^{k}\beta \,i\,S\,i$$

where *k*, β*_*i*_*, *S*_*i*_ represent the number of signature genes, the coefficient index, and the gene expression level, respectively.

To stratify patients into low- and high-risk groups, the optimum cutoff value for the risk score was determined using the “survminer” package in R. In order to ensure the comparability of the sample size between two groups, we set the *min.prop* parameter = 0.3 in applying the “survminer” package. Next, the Kaplan Meier survival curve and log-rank test was performed to evaluate the survival rates between low- and high-risk groups. The area under the receiver operating characteristic (ROC) curve (AUC) was calculated using the “survival ROC” package in R. In addition, the risk plot was illustrated using the “pheatmap” package in R.

### Identification of the Prognostic Factors for OS in Primary LGG

All patients with primary LGG in TCGA were randomly divided into the training and testing groups at a ratio of 7:3 using the “caret” package. Seven predominant clinical and prognostic factors, including age, gender, grade, radiotherapy, chemotherapy, IDH status, and the risk scores of the immune-related signature were evaluated using univariate and multivariate Cox regression analyses. Before that, we tested the proportional hazards assumption (Therneau, 1994) by Schoenfeld residuals analysis (Schoenfeld, 1982), using the statistical script language R (R Development Core Team, 2014). By employing “rms,” “foreign,” and “survival” R packages, we formulated a nomogram consisting of relevant clinical parameters and independent prognostic factors based on the multivariate Cox regression analysis. The performance of the prognostic nomogram was assessed by calculating Harrell’s concordance index (C-index) (Harrell et al., 1996), the AUC of the time-dependent ROC curve, and calibration curves of the nomogram for 3-, and 5-year OS plotted to estimate the accuracy of actual observed rates with the predicted survival probability. Time-dependent ROC analyses were conducted by “timeROC” R package.

### External Validation of the Signature in CGGA Datasets for Primary LGG

The prognostic capability of the immune-related risk signature was externally validated using CGGA database. The RNA-seq data and corresponding clinicopathological information were obtained from the CGGA database^3^. The specific risk score for each patient was calculated with the use of the prognostic gene signature. Similarly, patients were divided into low- and high-risk groups based on the constructed formula in TCGA database. The optimal cutoff of risk scores for CGGA dataset kept the same as that in primary TCGA cohorts. Survival curves for the low- and high-risk groups were plotted using Kaplan-Meier analysis. Next, the predictive accuracy of the signature was investigated using ROC curves, and the performance of the nomogram was also assessed by the time-dependent ROC curve and calibration.

### Investigation of the Signature in Patients With Recurrent LGG

For testing the prediction model in patients with recurrent LGG, the main inclusion criteria were: (1) patients suffering from recurrent glioma with histologically confirmed WHO II or III grade, (2) evidence of tumor recurrence and complete clinicopathological factors, (3) available recurrent glioma RNA-sequencing profiling, (4) minimum follow-up of 90 days. The exclusive criteria were as follows: (1) incomplete survival status and clinical information, (2) primary LGG samples. Time-dependent ROC curve and calibration plots were created to investigate whether the built model could effectively predict survival in recurrent LGG.

### Tumor-Infiltrating Immune Cell Analysis

To characterize the abundance of 22 immune cell types based on the RNA-seq data in lower grade glioma tissues, the CIBERSORTx web tool was applied^4^. Using a deconvolution algorithm (Newman et al., 2019), CIBERSORTx computed that the 22 cell types encompassed among others B cells, T cells, natural killer (NK) cells, macrophages, and dendritic cells (DCs). CIBERSORTx derived an empirical *P*-value for the deconvolution of each case using Monte Carlo sampling, and samples with *P* < 0.05 were adopted for analysis because of high reliability of the inferred cell composition (Ali et al., 2016). Therefore, cases with a *P* value of ≥0.05 were not retained for subsequent analysis. For validating the accuracy of the CIBERSORTx, TIMER (Tumor Immune Estimation Resource) database was also employed to illustrate the abundance of six immune cells containing B cells, CD4^+^ T cells, CD8^+^ T cells, macrophages, neutrophils, and dendritic cells^5^. Subsequently, the box plots were utilized to present the difference of infiltrative immune cells, T cell activated and inhibitory receptors, and macrophage associated molecules between high and low risk groups using the “ggplot2” package. In addition, the Cox regression model was also applied to calculate the hazard ratios (HRs) of the abundance of immune cells between high-and low-risk groups and illustrated by the forest plot.

### Validation of Gene Expression in Cell Lines and Glioma Tissues

The Cancer Cell Line Encyclopedia (CCLE) was generated to provide a compilation of mRNA expression, copy number variation, and preclinical datasets for mutations in various cancer types. Details regarding the acquisition of mRNA expression of six genes profiled by RNA-Seq were downloaded from the data portal^6^ (Barretina et al., 2012). The genomic data were utilized to analyze the mRNA expression status of the six immune genes in LGG cell lines. Cell lines of LGG were identified through six dedicated websites^7^ ^,8^ ^,9^ ^,10^ ^,11^ ^,12^. We only retained the consistent LGG cell lines across six websites. Furthermore, the level of protein expression for these six IRGs were confirmed using immunohistochemistry data publicly available at http://www.proteinatlas.org/. This database was explored to verify the gene-specific expression information across normal human tissues, as well as LGG.

### Statistical Analysis

All statistical analyses were conducted using R (version 3.6.0). The Wilcox test was used to screen statistically differentially expressed genes and infiltrative immune cells. Pearson’s chi-square tests were executed for the comparison of categorical variables. Kaplan–Meier curve using the log-rank test was used to evaluate the statistical significance of the survival rates between different risk groups. The predictive accuracy of the risk signatures were determined by ROC curves. The proportional-hazards assumption was tested with Schoenfeld residuals. Then, univariate and multivariate Cox regression analysis were performed to evaluate significantly prognostic factors. Finally, results of multivariate Cox regression analyses were visualized with nomogram. Concordance index, time-dependent ROC, and calibration were also important indicators used to assess the nomogram. *P* value < 0.05 was considered statistically significant.

## Results

### Preparation of Glioma Datasets

The workflow of our study is delineated in Supplementary Figure S1. A total of 916 patients who met the inclusion criteria, including 432 patients with primary LGG from the TCGA database, 353 patients with primary LGG from the CGGA database, and 131 patients with recurrent LGG from the CGGA database were obtained for further analysis. The clinicopathological characteristics of patients from the two databases are listed in Table 1.

**TABLE 1:** Summary of risk scores and clinical pathological characteristics for different cohorts.

	Primary LGG			
		Internal	External	Recurrent LGG
	Training	Validation	Validation	
	Cohort	Cohorts	Cohorts	Investigation
	TCGA	TCGA	CGGA	CGGA
Characteristic	(*n* = 304)	(*n* = 128)	(*n* = 353)	(*n* = 131)
**Age (y)^1^**				
≤40	152 (50%)	53 (41%)	189 (54%)	69 (53%)
>40	152 (50%)	75 (59%)	164 (46%)	62 (47%)
**Gender**				
Male	175 (58%)	62 (48%)	205 (58%)	76 (58%)
Female	129 (42%)	66 (52%)	148 (42%)	55 (42%)
**Grade**				
II	139 (46%)	66 (52%)	196 (56%)	32 (24%)
III	165 (54%)	62 (48%)	157 (44%)	99 (76%)
**Radiation**				
No	109 (36%)	47 (37%)	59 (17%)	26 (20%)
Yes	195 (64%)	81 (63%)	294 (83%)	105 (80%)
**Chemotherapy**				
No	134 (44%)	61 (48%)	147 (42%)	34 (26%)
Yes	170 (56%)	67 (52%)	206 (58%)	97 (74%)
**IDH^2^ status**				
Wild-type	53 (17%)	27 (21%)	94 (27%)	31 (24%)
Mutation	251 (83%)	101 (79%)	259 (73%)	100 (76%)
**Risk score**				
Low risk	209 (69%)	88 (69%)	248 (70%)	94 (72%)
High risk	95 (31%)	40 (31%)	105 (30%)	37 (28%)

*^1^Age, Age at pathological diagnosis of glioma; ^2^IDH, Isocitrate dehydrogenase.*

### Identification of DEGs

**FIGURE 1:**
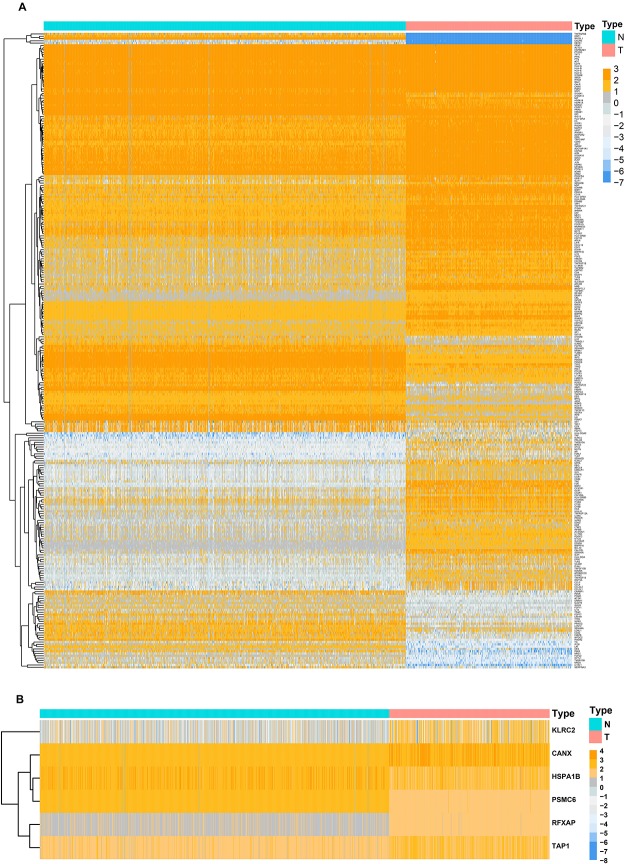
Heatmaps of differentially expressed genes between normal tissue and lower-grade glioma. **(A)** Heatmap demonstrating the differential expressed 277 immune-related genes. **(B)** Heatmap demonstrating the differential expressed six immune-related risk genes.

Before the identifying of DEGs, the normalization and batch effects removal from GTEx and TCGA datasets was conducted by “sva” package. As shown in Supplementary Figures S2A,C, the normalization of the data was performed well by the “sva” package. Additionally, the PCA plot found that TCGA and GTEx datasets separated obviously (Supplementary Figures S2B,D). To identify DEGs between the TCGA and GTEx databases, we considered the absolute value of the log2-transformed fold change (FC) > 1 and the adjusted *P*-value (adj.P) < 0.05 as the threshold levels of significance. Compared to non-tumor tissues, a total of 5,490 DEGs consisting of 2,718 upregulated and 2,772 downregulated genes were identified. The heatmap and volcano plot of the DEGs are shown in Supplementary Figures S3A,B. IMMPORT^13^ is a web server for acquiring immune gene lists. From this set of DEGs, a total of 277 differentially expressed IRGs were extracted. The heatmap of 277 differentially expressed IRGs was shown in Figure 1A.

### Identification of Prognostic IRGs

**FIGURE 2:**
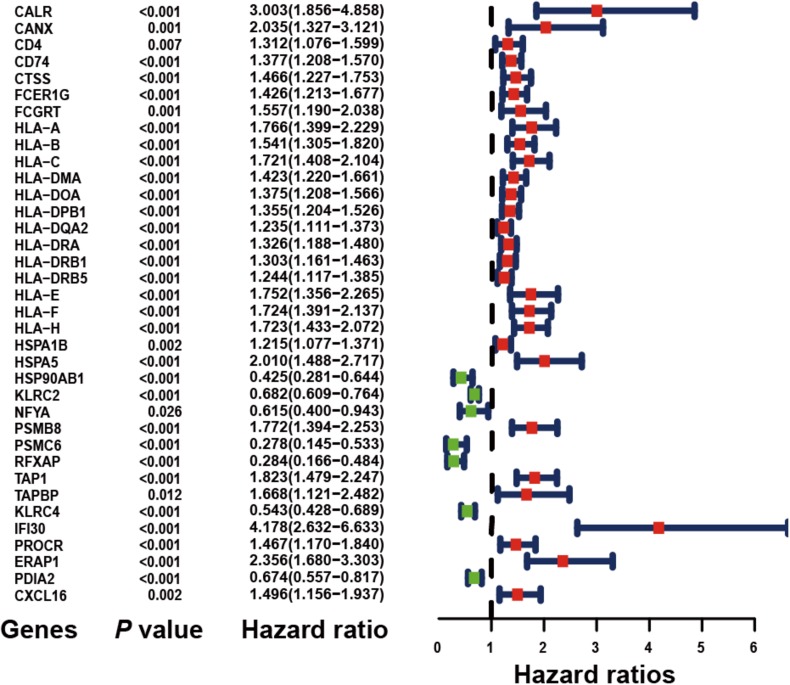
Forest plot of hazard ratios demonstrating the prognostic values of immune-related genes (IRGs). The dash line was used to mark the location of HR = 1. The red box represents the adverse prognostic factor; Blue box represents the favorable prognostic factor.

Based on the univariate Cox regression model (*P* < 0.05), a total of 36 IRGs were discovered to be significantly associated with OS. A forest plot of HR showed that 29 IRGs were risk factors, whereas 7 IRGs were protective factors (Figure 2).

### Evaluation of IRGs With Prognostic Value

Considering collinearity and following refinement by the Lasso, only 11 genes were remained in Lasso regression from 36 significant prognosis associated IRGs in univariate Cox regression model. Ultimately, a prognostic signature comprisingsix IRGs, including calnexin (*CANX*), heat shock protein family A (HSP70) member 1B (*HSPA1B*), killer cell lectin like receptor C2 (*KLRC2*), proteasome 26S subunit, ATPase 6 (*PSMC6*), regulatory factor X associated protein (*RFXAP*), and transporter 1, ATP-binding cassette subfamily B member (*TAP1*) was selected to construct a prediction model by stepwise multivariate Cox regression analysis. Correspondingly, the coefficients of the six genes were 0.38625, 0.18073, −0.27702, −0.71285, −0.68077, and 0.34100. Ultimately, the hazard ratios of the six genes were 1.4714, 1.1981, 0.7580, 0.4902, 0.5062, and 1.4064, respectively. The comprehensive risk score was imputed as follows: (0.38625 × expression level of *CANX*) + (0.18073 × expression level of *HSPA1B*) + (−0.27702 × expression level of *KLRC2*) + (−0.71285 × expression level of *PSMC6*) + (−0.68077 × expression level of *RFXAP*) + (0.34100 × expression level of *TAP1*). Optimal cutoff values for the risk scores were calculated using the “survminer” package. Thus, patients were stratified into low- (risk score < 1.28) and high-risk (risk score ≥ 1.28) groups. In addition, the differential expression of six risk genes between normal brain and LGG tissues were shown in Figure 1B.

### Performance of Risk Signature in Primary LGG From TCGA

**FIGURE 3:**
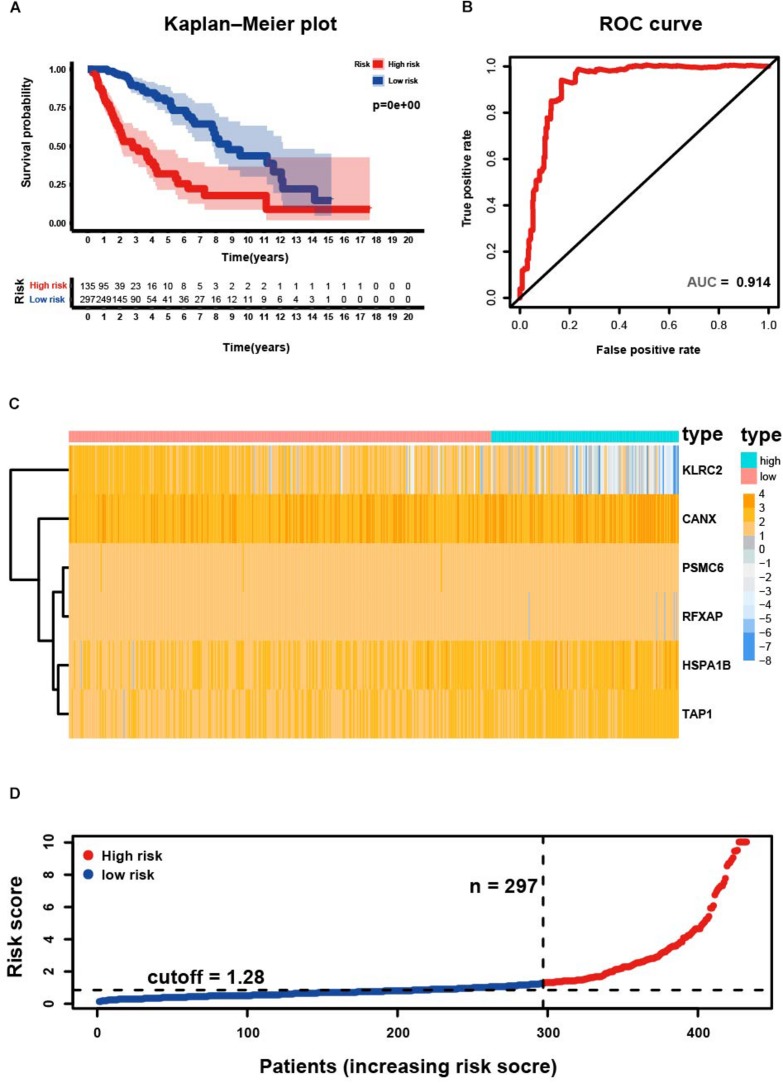
Development of risk score based on the six immune-related gene signature of patients with primary LGG in TCGA. **(A)** Kaplan-Meier plot for overall survival (OS) based on risk score of the six gene based signature of patients with primary LGG in the TCGA cohort. **(B)** ROC curve with an AUC of 0.914, indicating that risk score plays a significant performance in the efficacy of this diagnosis. **(C)** Heatmap demonstrating the distribution of the six immune-related gene expression in the TCGA cohort. **(D)** Risk plot presenting each point sorted based on risk score, representing one patient. Blue, and red represent patients with low- and high-risk, respectively.

Four hundred and thirty-two patients with primary LGG from the TCGA database were included in subsequent survival analyses and divided into low- and high-risk groups. Kaplan–Meier plots indicated that patients with high-risk scores presented a worse OS probability (Figure 3A). To verify the diagnostic competence of the immune-related risk signature, theAUC was calculated. The AUC of the ROC was 0.914, indicating that the risk score literally played a significant performance in the efficacy of this diagnosis (Figure 3B). The heatmap demonstrated that *KLRC2* exhibited the lowest expression in the high-risk group, whereas *CANX*, *HSPA1B*, *PSMC6*, *RFXAP*, and *TAP1* had medium and high expression levels (Figure 3C). Consecutively, patients appeared to have an increased mortality rate with an increase in risk scores according to the risk plot (Figure 3D).

### Construction of Prognostic Signature in Primary LGG From TCGA

**FIGURE 4:**
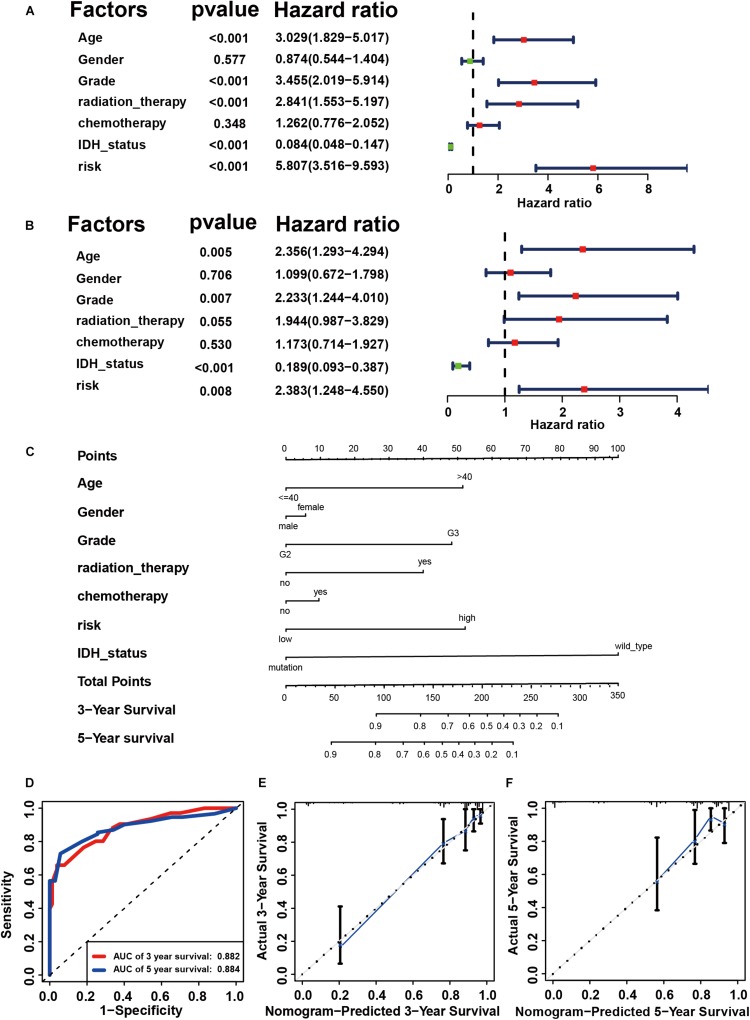
Construction of prognostic signature in primary LGG from TCGA. Univariate **(A)** and multivariable Cox proportion hazard regression for OS **(B)** of primary LGG in training group. **(C)** A nomogram consisting of risk score and other clinical indicators for predicting 3-, and 5-year OS of primary LGG. **(D)** Time-dependent ROC for 3-, and 5-year OS predictions for the nomogram compared with actual observations. Calibration plot of nomogram for predicting probabilities of 3-year **(E)**, and 5-year **(F)** overall survival of patients. Blue line indicates actual survival.

Using the “caret” package, the 432 patients with primary LGG in the TCGA dataset were randomly separated into training and testing cohorts at a ratio of 7:3. Seven clinicopathological parameters recorded as binary variables: age (≤40 vs. >40), gender (male vs. female), grade (grade II vs. grade III), radiotherapy (yes vs. no), chemotherapy (yes vs. no), risk (low vs. high), and IDH status (wild-type vs. mutation) were employed into further analyses, following testing of the proportional hazards assumption with Schoenfeld residual plots (Supplementary Figure S4). To evaluate the independent prognostic force of the signature, both the univariable and multivariable Cox proportion hazard regression models were applied (Figures 4A,B). Results from univariable analysis showed that risk (HR = 5.807, *P* < 0.001), age (HR = 3.029, *P* < 0.001), grade (HR = 3.455, *P* < 0.001), radiation therapy (HR = 2.841, *P* < 0.001), and IDH status (HR = 0.084, *P* < 0.001) had prognostic value for OS in primary LGG. Likewise, the risk group (HR = 2.383, *P* = 0.008), age (HR = 2.356, *P* = 0.005), grade (HR = 2.233, *P* = 0.007) and IDH status (HR = 0.189, *P* < 0.001) maintained their prognostic values in multivariable stepwise cox regression analysis. Next, risk, age, gender, grade, radiotherapy, chemotherapy, and IDH status were visualized in the nomogram. Nomograms of 3- or 5-year OS in the cohort are presented in Figure 4C. Then, the C-index for the training group was 0.8642. The AUC of the nomogram was up to 0.88, indicating the excellent ability to discriminate patients of poor from patients of favored prognosis (Figure 4D). Meanwhile, the calibration curve also manifested a satisfactory agreement between predictive and observational values at the probabilities of 3- and 5-year survival (Figures 4E,F). These results revealed that the nomogram signified good accuracy in predicting the 3- or 5-year survival of patient with LGG.

### Internal Validation of Prognostic Signature in Primary LGG From TCGA

**FIGURE 5:**
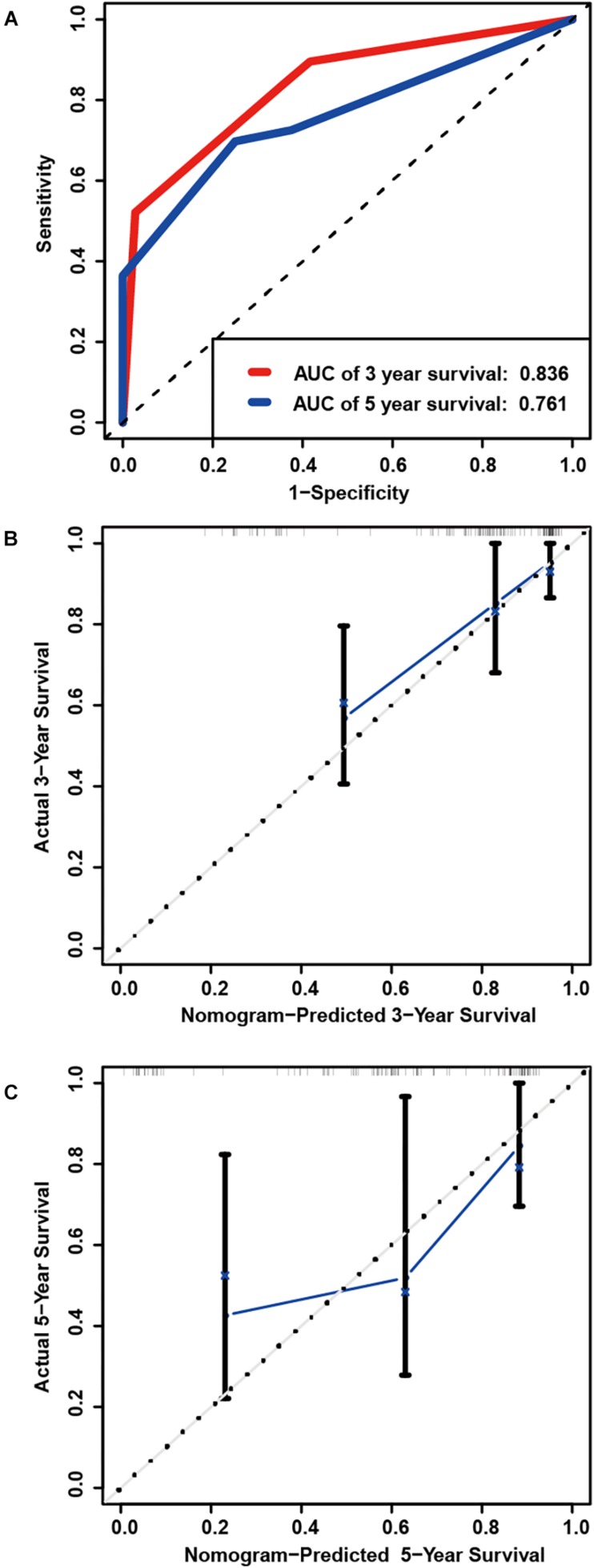
Internal validation of prognostic signature in primary LGG from TCGA. **(A)** Time-dependent ROC curve based on the six genes based risk score for 3-, and 5-year OS probability in the internal validation cohort. Calibration plot for internal validation of 3-year **(B)**, and 5-year **(C)** OS of patients.

A total of 128 patients with primary LGG in the TCGA dataset were randomly assigned in the internal cohort and the predictive power of the signature was accordingly confirmed. Each of the cases was divided into low- and high-risk groups. The C-index for the internal validation group was 0.8309. Time-dependent ROC analyses at 3- and 5-year were conducted to assess the prognostic accuracy of the six-gene-based classifier. The 3- and 5-year AUC were 0.836 and 0.761, respectively (Figure 5A). The calibration curve also manifested a satisfactory agreement between predictive values and observational values at the probabilities of 3- and 5-year survival (Figures 5B,C).

### External Validation of Prognostic Signature in Primary LGG From CGGA

**FIGURE 6:**
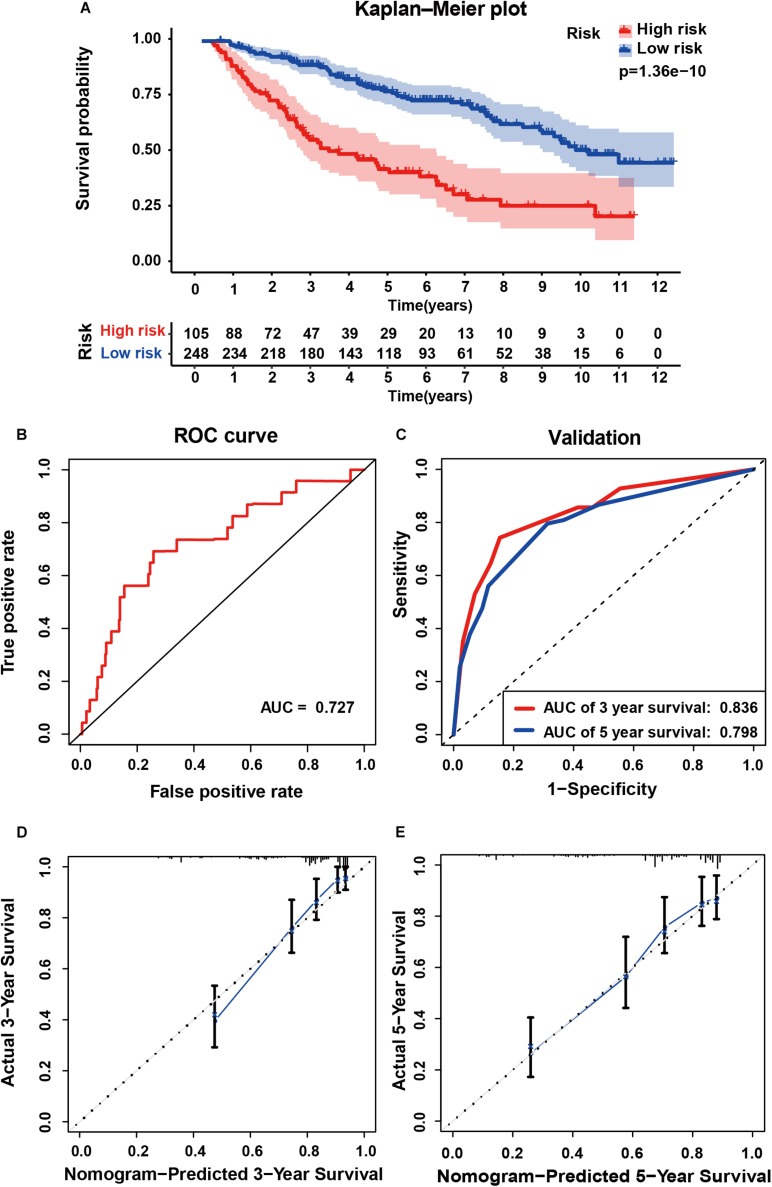
External validation of the six gene signature in primary LGG inform the CGGA dataset. **(A)** Kaplan-Meier survival curves of the six gene signature of patients with primary LGG in the CGGA cohort. **(B)** ROC curve for assessing diagnostic competence of the risk score in the CGGA cohort. **(C)** ROC curves for 3-, and 5-year OS predictions for the six gene signature in the external validation cohort. Calibration curves for predicting probabilities of 3-year **(D)**, and 5-year **(E)** OS of patients in external validation.

To determine whether the six-gene prognostic signature had similar prognostic value in different populations, its prediction performance was validated in another 353 primary LGG samples with RNA-seq transcriptome data and corresponding clinicopathological information from the CGGA database. The primary LGG samples were divided into two groups according to the cutoff value (<1.28 vs. ≥1.28). Consistent with the above findings, the Kaplan-Meier survival curves revealed a significant difference in OS between the low- and high-risk groups (Figure 6A). The AUC was 0.727, showing a fair prognostic power of the model (Figure 6B). To evaluate the prognostic accuracy of the model, time-dependent ROC analysis was conducted, with the AUC for 3, and 5-year survival being 0.836 and 0.798, respectively (Figure 6C). The C-index for the CGGA group was 0.7555. The calibrations plot for survival probability at 3- or 5-year showed an optimal consensus between the prediction and observation in both the external validation and training cohorts (Figures 6D,E).

### Investigating the Application of Six Genes Based Signature in Recurrent LGG

Next, we investigated the feasibility of the six -immune-gene related risk signature in recurrent LGG. According to inclusive and exclusive criteria, 131 patients with recurrent LGG were enrolled for further analysis. Risk scores were calculated using the same formula and yielded similar results on Kaplan-Meier survival curves as those observed for primary LGG (*P* < 0.05; Supplementary Figure S5A). However, the AUC value was only 0.550, indicating a poor prognostic power in recurrent LGG (Supplementary Figure S5B). The C-index for the recurrent LGG group was 0.6135. Then, the AUC for 3-, and 5-y OS predictions for the recurrent cohort was 0.631, and 0.638, respectively (Supplementary Figure S5C). Meanwhile, the verification of the recurrent LGG cohort using the calibration plot was not satisfactory (Supplementary Figures S5D,E).

### The Association Between Risk Score and Clinicopathological Parameters

Subsequently, we analyzed the relationship between the six-gene signature and clinicopathological parameters (age, gender, grade, radiotherapy, chemotherapy, and IDH mutation status) in LGG. In terms of grade and IDH status, patients of grade III or of the IDH wild type had higher risk scores than those with grade II or of the IDH mutant type, consistent with the findings in patients with primary LGG from CGGA. Moreover, data of patients with primary glioma from TCGA revealed that older patients had significantly higher risk scores than those of younger. Risk scores were also comparable across recurrent LGG in CGGA, with results revealing a preference for higher levels of risk scores in males. However, no significant difference was observed between the IDH wild and mutant groups in recurrent LGG (Supplementary Figures S6A,C).

### Correlation of the Risk Score With Tumor-Infiltrating Immune Cells

**FIGURE 7:**
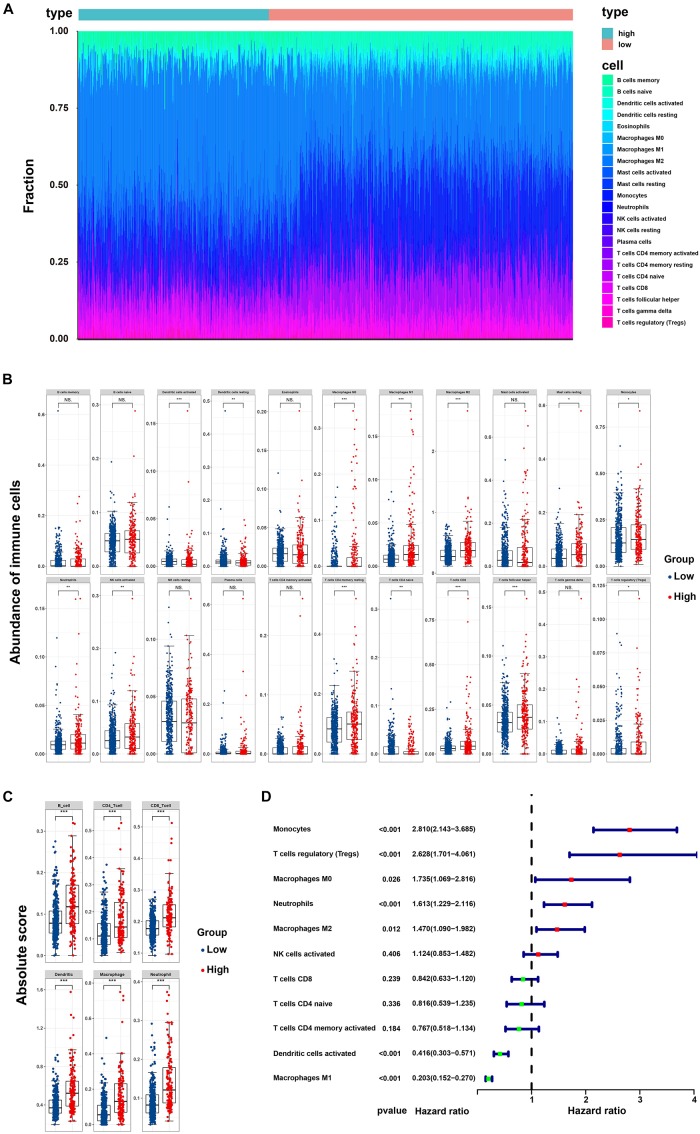
Correlation of the risk score with infiltrative immune cells. **(A)** Barplot showing the percentages of 22 infiltrative immune cells calculated by CIBERSORTx between high-and low-risk groups in primary LGG from TCGA and CGGA cohorts (high-risk, 232 samples; low-risk, 551 samples); **(B)** Boxplot showing the differential abundance of 22 infiltrative immune cells calculated by CIBERSORTx between high-and low-risk group in primary LGG; **(C)** Boxplot showing the differential abundance of six infiltrative immune cells by TIMER database between high-and low-risk group in primary LGG; **(D)** Forest plot of hazard ratios demonstrating the prognostic values of 22 immune cells calculated by CIBERSORTx in primary LGG. The dash line was used to mark the location of HR = 1. The red box represents the adverse prognostic factor, and the blue box represents the favorable prognostic factor.

By applying the CIBERSORTx algorithm to RNA-seq data, the relative proportions of 22 immune cell subsets of LGG were acquired. Consecutively, 432 cases of primary LGG in the TCGA dataset, 351 cases of primary LGG in the CGGA dataset were enrolled for further analysis after the filter criteria with *P* value < 0.05 via CIBERSORTx algorithms. As shown by bar plot in Figure 7A, the abundance of the 22 infiltrative immune cells by using CIBERSORTx were significantly different between high-risk and low-risk groups in primary LGG cohorts. Among them, the macrophage M2 was the most significant enrichment of immune cells. Subsequently, as shown in the box plots (Figure 7B), the infiltration levels of CD8^+^T cells, resting memory CD4^+^T cells, follicular helper T cells, regulatory T cells, activated NK cells, monocytes, macrophages (M0, M1, M2), activated DCs, resting mast cells, and neutrophils were significantly higher in high-risk group than that in low-risk group. On the contrary, the infiltration levels of naïve CD4^+^T cells, and resting DCs were significantly higher in low-risk group. The differential abundance of the 22 infiltrative immune cells were summarized in Table 2. Furthermore, to validate the infiltrative abundance of immune cells in CIBERSORTx, the TIMER database was enrolled. As shown in Figure 7C, the B cells, CD4^+^T cells, CD8^+^T cells, DCs, macrophages, and neutrophils were all significantly higher in the high-risk group. To further investigate the prognostic values of the infiltrative immune cells, the univariate Cox proportion hazard regression models were applied. Results from Cox regression analysis showed that high abundance of Tregs, neutrophils, M2-type macrophages were significantly associated with unfavorable survival outcome (*P* < 0.001, *P* < 0.001, *P* = 0.012, respectively). Conversely, high abundance of macrophage M1 (HR = 0.203, *P* < 0.001), and activated DCs (HR = 0.416, *P* < 0.001) were identified as the protective factors in primary LGG (Figure 7D).

**TABLE 2:** The differential abundances of 22 infiltrative immune cell types between high-and low-risk groups of with primary LGG as calculated by CIBERSORTx.

	Mean	Mean		
Immune cell type	(high risk)	(low risk)	Difference	*P* value
B cells naive	0.050	0.045	0.005	0.126
B cells memory	0.021	0.018	0.003	0.819
Plasma cells	0.015	0.007	0.008	0.179
T cells CD8	0.073	0.035	0.037	0.000
T cells CD4 naive	0.007	0.010	–0.003	0.001
T cells CD4 memory resting	0.107	0.083	0.024	0.001
T cells CD4 memory activated	0.011	0.006	0.004	0.539
T cells follicular helper	0.040	0.034	0.007	0.001
T cells regulatory (Tregs)	0.008	0.005	0.003	0.016
T cells gamma delta	0.016	0.007	0.009	0.167
NK cells resting	0.029	0.030	–0.001	0.298
NK cells activated	0.040	0.030	0.009	0.009
Monocytes	0.170	0.153	0.017	0.033
Macrophages M0	0.025	0.007	0.018	0.000
Macrophages M1	0.024	0.010	0.013	0.000
Macrophages M2	0.415	0.306	0.109	0.000
Dendritic cells resting	0.015	0.017	–0.003	0.001
Dendritic cells activated	0.006	0.005	0.000	0.000
Mast cells resting	0.055	0.039	0.015	0.021
Mast cells activated	0.069	0.054	0.015	0.170
Eosinophils	0.020	0.017	0.003	0.673
Neutrophils	0.016	0.011	0.005	0.001

In addition, we also investigated the differential expressions of the T-cells activated and inhibitory receptors, and macrophage associated molecules between the high and low risk groups. As shown in Supplementary Figure S7, the T cells activation associated genes containing *CD40L*, *GITR*, *4-1BB*, *OX40*, *CD27*, *ICOS*, and *CD28* were significant higher in high-risk group. T cells inhibition associated genes containing *CTLA4*, *PD-L1*, *PD-1*, *CD80*, *CD244*, *TIM3*, *BTLA*, *CD160* were also significant higher in high-risk group. Moreover, macrophage chemo-attractant and phagocytosis related genes containing *CSF1*, *CSF1R*, *CCL2*, *CCR2*, and *CXCR4* were also significant higher in high-risk group.

### Six Genes Based Signature Expression Analysis in Databases

The expression of the six genes were queried from CCLE^14^. Results were sorted according to tumor type. The mRNA expression of *CANX*, *HSPA1B*, *PSMC6*, and *TAP1* was high in gliomas, whereas that of *KLRC2* was low (Supplementary Figures S8A–F). The expression of the six genes in 14 LGG cell lines is illustrated in Table 3. The Human Protein Atlas database was used to explore the protein expression levels of these six genes and results are shown in Supplementary Figure S9.

**TABLE 3:** List the expression of the six genes in 14 LGG cell lines.

	Gene expression (TPM)						
Cell lines	*CANX*	*HSPA1B*	*KLRC2*	*PSMC6*	*RFXAP*	*TAP1*	*RRID*
H4	448.522	22.6035	0.0220068	15.7311	2.2642	34.2997	CVCL_1239
HS683	355.23	15.2679	0.278926	14.4031	3.26084	51.7626	CVCL_0844
KG1C	363.096	13.9961	0.113268	18.5451	3.64382	26.4942	CVCL_2971
LN215	288.476	26.108	2.48677	14.8853	4.68699	87.4974	CVCL_3954
LN235	235.629	27.8249	0.0447866	17.1381	4.57199	19.3359	CVCL_3957
LN319	271.245	21.0797	0	20.656	3.41301	17.9863	CVCL_3958
LNZ308	169.63	17.2783	0	22.7476	3.24888	17.519	CVCL_0394
NMCG1	264.062	16.9103	0.0049317	14.6614	2.81961	37.3184	CVCL_1608
SF268	272.357	27.68	0.0245476	9.43665	1.16183	31.9634	CVCL_1689
SNU738	144.254	17.2946	0.0770556	12.636	1.80947	31.244	CVCL_5087
SW1088	290.749	23.2654	0.0473503	17.416	4.36785	29.0014	CVCL_1715
SW1783	351.565	27.6688	0.0224892	18.6383	2.55953	33.8329	CVCL_1722
TM31	134.804	7.78124	0.0414412	21.8484	5.08505	60.5928	CVCL_6735
U178	220.836	8.10508	0.552537	17.0014	1.73231	37.2859	CVCL_A758

## Discussion

Emerging evidence has demonstrated that the immune microenvironment plays an essential role in tumor biology, and recently, numerous inspiring clinical trials have established the role of immunotherapy in gliomas. Thus, immune related biomarkers show great potential in risk stratification and in exerting prognostic value. In previous studies, immune-gene related signatures have been identified as independent prognostic factors in several solid tumors (Ascierto et al., 2012; Kim et al., 2018; Shen et al., 2019; Yang et al., 2019), revealing that the immune status and local immune response could be illustrated by the risk signatures employed. However, the prognostic value and the association between immune status and risk signatures have not been fully elucidated in LGG. In the current study, 277 immune-related DEGs were identified. After Lasso regression and multicox analysis, six immune genes (*CANX*, *HSPA1B*, *KLRC2*, *PSMC6*, *RFXAP*, and *TAP1*) were identified as components of the risk signature to divide LGGs into low- and high-risk groups. Subsequently, KM curve, ROC curve and risk plot analyses verified that the six-based risk signature performs well in stratifying the risk groups of primary LGG in TCGA and CGGA datasets. Furthermore, in univariable analysis, the risk group, age, grade, radiation therapy and IDH status exhibited their predictive value regarding OS in primary LGG. Correspondingly, in multivariable stepwise cox regression analysis, with the exception of radiation therapy showing borderline significance, all other factors retained their prognostic values. Consecutively, it was found that the prognostic signature showed fair accuracy regarding the 3- and 5-year OS in the internal (TCGA) and external (CGGA) validation cohorts. However, predictive performance was poor in the recurrent LGG cohort.

At first, it was shown that the IRG-based risk signature could function as a proper index in stratifying risk groups in LGG. Similar to our study, Shen et al. (2019) also found that an immune gene based signature could significantly stratify patients into different risk groups in ovarian cancer. Correspondingly, another study also revealed that the immune-related gene signature was capable of stratifying patients into responder and non-responder groups in human breast cancer, with the odds ratios of the immune-related risk signature making it the most significant predictor of pathological complete remission (odd ratio: 4.6, 95% confidence interval: 2.7 to 7.7, *P* < 0.001) (Sota et al., 2014). Second, we found that the risk group, age, grade, radiation therapy and IDH status had predictive values for OS in primary LGG. According to National Comprehensive Cancer Network guidelines, the prognostic values of age (≤40 years vs. >40 years), tumor grade (II vs. III), and IDH status (wild-type vs. mutation) have been well-established in clinical practice (National Comprehensive Cancer Network, 2019). Compared with the above mentioned well-established clinicopathological prognostic factors, the risk group remained an independent prognostic value in univariate and multivariate cox regression analysis. In accordance with the present findings, Qian et al. (2018) also found that patients identified as high-risk by the IDH associated immune signature exhibited unfavorable prognosis in LGGs. The prognostic value of the local immune signature was also verified in glioblastomas. Risk scores were significantly associated with poor OS and progression-free survival (Cheng et al., 2016). Surprisingly, receiving or not radiation therapy was associated with OS in univariate analysis, but the relationship was borderline significant in multivariate analysis. In addition, the prognostic value of chemotherapy was also insignificant in our analysis. Our result is likely to be related to the undefined timing of radiation therapy (postoperative or palliative treatment), and differences in radiation dose or frequency. To our knowledge, numerous trials have investigated the prognostic values of chemotherapy and radiotherapy in gliomas, as well as their significant contribution in improving survival. The RTOG 9802 trial evaluated radiotherapy followed by adjuvant procarbazine, CCNU, and vincristine (PCV) chemotherapy in 251 patients with low-grade glioma and showed an improvement in median OS with the addition of PCV from 7.8 to 13.3 years (HR = 0.59; *P* = 0.002) (van den Bent, 2014). In the CATNON trial, the 5-year survival in patients with anaplastic glioma receiving combined chemo-radiotherapy was significant higher than that in patients receiving radiotherapy alone (55.9 vs. 44.1%, HR = 0.65; *P* = 0.0014) (van den Bent et al., 2017). The lack of prognostic values of chemotherapy and radiotherapy in our study, might be owing to several reasons: (1) undefined chemotherapy strategy (pre-radiotherapy or concurrent or adjuvant chemotherapy); (2) undefined chemotherapy regimens in the TCGA datasets; (3) undefined radiation regimens (postoperative or palliative treatment strategy, differences in radiation dose or frequency). Therefore, new trials are encouraged to further develop and verify our risk signature in standard treatment cohorts.

Emerging evidence have confirmed the prognostic values of immune genes in various cancers (Patel et al., 2013; Surmann et al., 2015; Yang et al., 2015; Ling et al., 2017; Ding et al., 2018). In current study, six IRGs were identified as the risk signature. Among them, *CANX*, *HSPA1B*, and *TAP1* were shown to be risk-associated genes, whereas *KLRC2*, *PSMC6*, and *RFXAP* were identified as protective genes. They have been reported to be involved in the regulation of immune response. Calnexin, an essential endoplasmic reticulum (ER) chaperone protein, plays a vital role in the synthesis of HLA class I surface antigen complex. Calnexin was revealed to inhibit the proliferation and activation of CD4^+^T and CD8^+^T cells, and it may impair the function of T cells by upregulating the expression of PD-1 in oral squamous cancer (Chen et al., 2019). Consistent with our results, it was found that decreased expression of *CANX* was associated with favorable survival outcome (Patel et al., 2013) and served as a biomarkers for tumor response in glioblastoma (Demeure et al., 2016). TAP1, an essential component of the major histocompatability complex (MHC) class I antigen-presenting pathway. It was found to be associated with tumor immune escape and prognosis (Leone et al., 2013). Ling et al. (2017) found that the expression of *TAP1* was significantly associated with infiltrative general T cells (CD3^+^), CD8^+^ cytotoxic T cells, M1-type macrophages, and M2-type macrophages, and the expression of *TAP1* could serve as an independent prognostic factor in colorectal cancer. In term of HSP70, encoded by *HSPA1B*, has emerged as a promising antitumor target in various cancer. Recently, it is also revealed that HSP70 may serve as a diverse immunoregulatory factors by acting as a cytokine in antigen presentation, DC maturation, the activities of NK cells, and myeloid-derived suppressor cells (Jego et al., 2019). Correspondingly, it was illustrated that up-regulation of *HSPA1B* was associated with poor outcomes in hepatocellular carcinoma (Yang et al., 2015). Comparatively, the investigations of *KLRC2* in cancer research is rare. To our knowledge, as a transmembrane activating receptor in NK cells, *KLRC2* is expressed in most NK cells and subsets of CD8^+^T cells (Wischhusen et al., 2005; Borrego et al., 2006). PSMC6, as a critical component of 26S-proteasome complex, involving in numerous pathways: antigen presentation (Livneh et al., 2016), cell proliferation and migration (Guo and Dixon, 2016). Zhu et al. (2018) demonstrated that PSMC6 may involve in the downstream of silencing cat eye syndrome critical region protein-1 in targeting the proliferation of TAM in glioma. *RFXAP*, as a vital transcription factor for major histocompatibility complex (MHC) class II. It was revealed to downregulate the expression of MHC class II in DCs (Ding et al., 2015) and macrophages (Wu et al., 2019), resulting inhibition of CD4^+^T cells infiltration (Surmann et al., 2015). It was associated with survival outcomes in solid tumors (Surmann et al., 2015; Ding et al., 2018). Overall, the prognostic values of the six risk genes have been exploited in various cancers, and their contribution to immune regulations were mainly concentrated on antigen presenting cells and effector T lymphocytes. Hence, further investigation is warranted to illustrate the correlations between risk groups and infiltrative immune cells in primary LGG.

The immune microenvironment has been identified as playing a critical role in tumor biology (Hanahan and Weinberg, 2011). Numerous studies have exploited the critical roles of infiltrative immune cells in glioma (Perus and Walsh, 2019; Wang et al., 2020). In current study, it was found that the M2-type macrophage was significantly enriched in primary LGG. Despite the glioma was defined as “cold tumor” with very little infiltrative immune cells, the proportions of macrophage can still constitute up to 30–50% in the TME of glioma (Guadagno et al., 2018). Additionally, the predictive values of immune cells have been extensively investigated. It was demonstrated that high levels of M2-type macrophages (marked as CD204 or CD206) (Ding et al., 2014), neutrophils (Liang et al., 2014), Tregs (Iwata et al., 2019) were defined as the adverse prognostic factors in glioma. Conversely, high levels of M1-type macrophages (Ding et al., 2014), CD8^+^T cells (Kmiecik et al., 2013) were identified as protective factors in glioma. Likewise, our results also revealed that elevated abundance of M2-type macrophages, neutrophils, and Tregs were associated with adverse survival outcomes. On the contrary, increased abundance of M1-type macrophages, and CD8^+^T cells were associated with favorable survival outcomes. As mentioned above, the six risk genes can not only have intrinsic roles in tumor growth and apoptosis (i.e., Guo and Dixon, 2016; Chen et al., 2019; Jego et al., 2019), but also serve as the immune-regulatory factors via antigen-presenting cells (APCs) and effector T lymphocytes (Borrego et al., 2006; Surmann et al., 2015; Ling et al., 2017; Zhu et al., 2018; Chen et al., 2019; Wu et al., 2019). Hence, it is worthwhile to explore the relationship between the risk groups and infiltrative immune cells in primary LGG. Interestingly, it was found that the abundance of macrophages, activated DCs, NK cells, CD8^+^T cells were significantly higher, while that of naïve CD4^+^T cells were significantly lower in high-risk group. Moreover, our results also demonstrated that high riskscores were associated with aggressive tumor subtypes, rapid proliferation and shorter survival time. Therefore, we hypothesized that malignant proliferation in high-risk patients may be accompanied with elevated tumor mutation burden and increased necrosis and apoptosis, which lead to continuous exposure of neoantigens and subsequent activation of the immune response. Consequently, high levels of infiltrative APCs and effector cells (including NK, CD4^+^T, and CD8^+^T) were observed in TME of primary LGG. Correspondingly, our results in Supplementary Figure S6 also illustrated that macrophage associated chemo-attractant molecules and T cell activating receptors were significant higher in high-risk group. Meanwhile, as a compensation response to increased immune activation (Perus and Walsh, 2019), the expressions of inhibitory molecules containing CTLA-4, PD-1, PD-L1, TIM-3, etc. (Wherry and Kurachi, 2015) were relatively higher in high-risk group. Noteworthy, it is necessary to clarify the positive relationship between riskscores and increased infiltrative immune cells. The aggressive phenotypes determined by the dysregulation of the six risk genes was fluctuated with the proportions of immune cells in TME, indicating that these genes may involve in the process of neoantigen presence and trigger the immune response. Considering that tumor cell is the large group of the antigen-presenting cells, 14 LGG cells lines were employed to validate the expression of six risk genes. It is obvious that all the six risk genes were commonly expressed, even some were high expressed in LGG cell lines. Further *in vivo* and *in vitro* experiments are warranted to investigate the mechanisms of six genes in LGG and the communications with immune cells in TME.

Our study, however had several limitations that should be addressed. First, because of the retrospective design and despite strict inclusive and exclusive criteria, selection and recall bias are unavoidable; Second, due to lack of complete chemotherapy and radiotherapy regimens in the current study, their prognostic values could not be fully elucidated. Third, although the 1p19q codeletion status constitutes a vital prognostic factor in clinical practice, such information was unavailable in the TCGA datasets and hence, was not employed in our prognostic signature. Fourth, although the six-based genes risk signature indicated a fair predictive ability for 5-year survival, more key factors are still needed to be brought into analysis. This is owing to the poor performance in predicting the survival outcome in recurrent LGG. Thus, it is reasonable to aim to utilize more factors into building a prognostic model that could enable risk stratification of recurrent LGG. Fifth, as molecular mechanism have not been investigated in the current study, it is necessary to explore the underlying mechanisms behind the risk scores and poor survival outcomes of LGG in further *in vitro* or *in vivo* experiments. Sixth, the “sva” package was applied in current study to remove the batch effects of Level 3 data from TCGA and GTEx. Despite the two groups separated obviously, however, several outliers can be found in the PCA plots. It should be noted that the reasons of several outliers may be caused by the insufficient batch effect removal of Level 3 data by “sva” (Wang et al., 2018) or others such as different parts of brain tissues or lacking reference of normal controls in TCGA, all of them warranting further investigations.

## Conclusion

In this study, we demonstrated that a six immune-related genes based risk signature might be effective in risk stratification and in serving as an independent prognostic factor of the overall survival in patients with primary LGG. Further *in vitro* and *in vivo* experiments are warranted to explore the underlying mechanisms behind immune genes and survival outcome in primary LGG.

## Data Availability Statement

Publicly available datasets were analyzed in this study. The RNA-seq data (level 3) and clinical information of LGG samples can be found in UCSC Xena (http://xena.ucsc.edu/), and the CGGA database (http://www.cgga.org.cn). The immune-related genes available at https://immport.niaid.nih.gov. The mRNA expression of genes profiled by RNA-Seq available at https://portals.broadinstitute.org/ccle.

## Ethics Statement

All the information of patients was obtained from Chinese Glioma Genome Atlas (CGGA), and The Cancer Genome Atlas (TCGA). All the patients and treatments were complied with the principles laid down in the Declaration of Helsinki in 1964 and its later amendments or comparable ethical standards.

## Author Contributions

MZ analyzed the data. MZ, XW, and JH contributed materials or analysis tools. XW prepared the figures and tables. MZ, XC, and XW authored or reviewed drafts of the manuscript. QZ and JH conceived and designed the study. QZ revised the manuscript.

## Conflict of Interest

The authors declare that the research was conducted in the absence of any commercial or financial relationships that could be construed as a potential conflict of interest.

## Footnotes

1 http://xena.ucsc.edu/

2 https://immport.niaid.nih.gov

3 http://www.cgga.org.cn

4 https://cibersortx.stanford.edu/

5 https://cistrome.shinyapps.io/timer/

6 https://portals.broadinstitute.org/ccle

7 https://web.expasy.org/cellosaurus/

8 https://www.atcc.org/

9 https://www.phe-culturecollections.org.uk/products/celllines/generalcell/search.jsp

10 http://igrcid.ibms.sinica.edu.tw

11 https://cansarblack.icr.ac.uk/

12 https://www.dsmz.de/

13 http://immport.org

14 https://portals.broadinstitute.org/ccle

## Supplementary Material

FIGURE S1 The flowchart of the project.

FIGURE S2 The normalization and batch effect removal from TCGA and GTEx datasets. **(A)** Box plots illustrated the data distributions from TCGA and GTEx datasets before normalization. **(B)** PCA plot illustrated the cluster of the samples from TCGA and GTEx datasets before batch effect removal. **(C)** Box plots illustrated the data distributions from TCGA and GTEx datasets after normalization. **(D)** PCA plot illustrated the cluster of the samples from TCGA and GTEx datasets after batch effect removal.

FIGURE S3 **(A)** Heatmaps showing that the 5,490 differentially expressed genes (DEGs) can effectively distinguish tumors from non-tumor tissues after integrated analysis. **(B)** Volcano plot presenting DEGs between LGG and non-tumor tissues. Red dots, and green dots represent up-regulated genes, and down-regulated genes, respectively.

FIGURE S4 Schoenfeld residual plots showing *P* value of all factors were greater to 0.05.

FIGURE S5 Investigating the application of six genes based signature in recurrent LGG. **(A)** Kaplan-Meier plot for overall survival based on risk score of the six gene based signature of recurrent LGG patients in CGGA cohort. **(B)** ROC curve based on the risk score for diagnostic competence verification of recurrent LGG patients in CGGA cohort. **(C)** Time-dependent ROC curve based on the six genes based risk score for 3-, and 5-year overall survival probability of recurrent LGG patients in CGGA cohort. Calibration curve for predicting probabilities of patients’ 3-year **(D)**, and 5-year **(E)** overall survival of recurrent LGG patients in CGGA cohort.

FIGURE S6 Association between risk score and clinical-pathological parameters. Association between risk score and age, gender, grade, radiotherapy, chemotherapy, and IDH mutation status of primary LGG patients in TCGA cohort **(A)**, in CGGA cohort **(B)**, while patients of recurrent LGG patients in CGGA cohort are shown in **(C)**.

FIGURE S7 The differential expressed T cell associated activated and inhibitory genes, macrophage chemo-attractant and phagocytosis related genes between high and low risk groups in primary LGG.

FIGURE S8 Expression data were sorted by the tumor type. The expression of the CANX **(A)**, HSPA1B **(B)**, KLRC2 **(C)**, PSMC6 **(D)**, RFXAP **(E)**, and TAP1 **(F)** in Cancer Cell Line Encyclopedia.

FIGURE S9 Number of patients with staining **(A)**. The typical protein expression of six genes of immunohistochemistry (IHC) images in LGG tissue and paired non-tumor samples **(B)**. Data was queried from the human protein atlas (https://www.proteinatlas.org/).
