# Supplementary figures and images for: Novel Immune-Related Gene Signature for Risk Stratification and Prognosis of Survival in Lower-Grade Glioma
Source: Front Genet. 2020 Apr 15;11:363. doi: 10.3389/fgene.2020.00363 (PMC7174786; doi:10.3389/fgene.2020.00363)

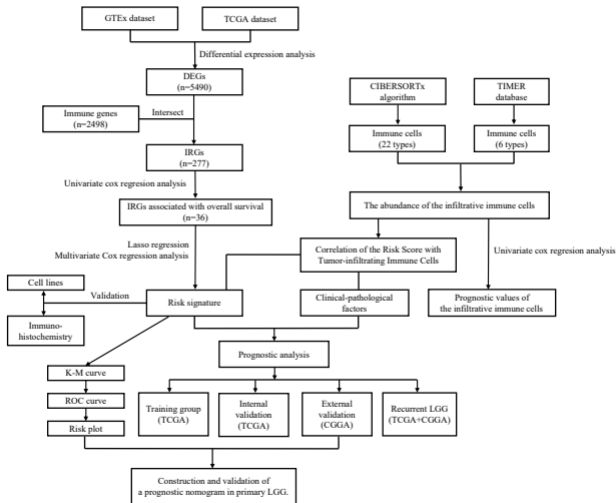

Supplement: FIGURE S1 — The flowchart of the project. [file Image_1.PDF]

**A**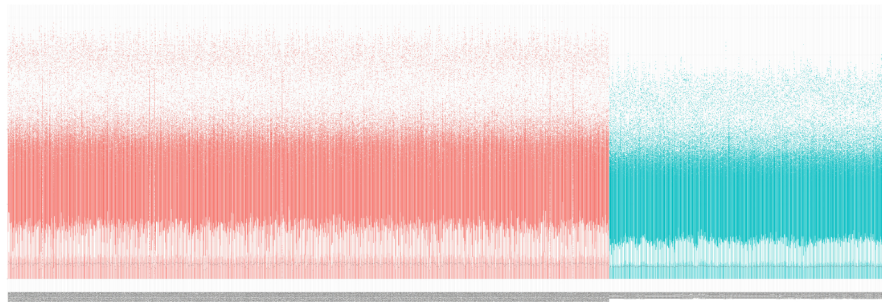**B**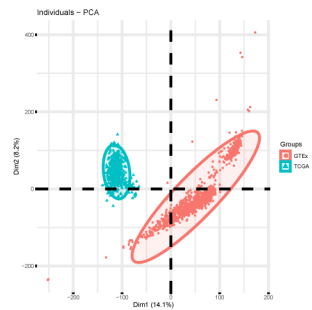**C**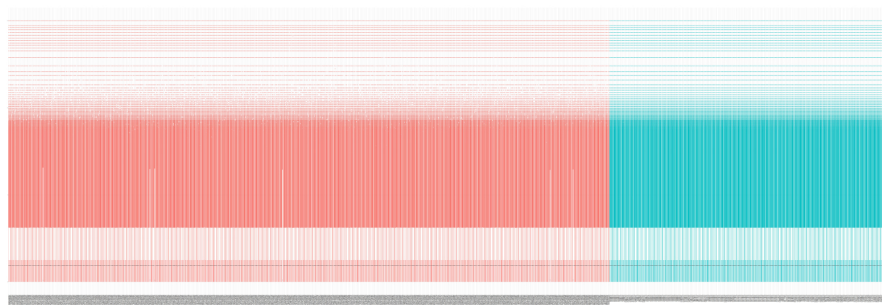**D**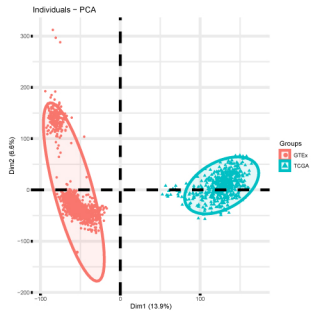

Supplement: FIGURE S2 — The normalization and batch effect removal from TCGA and GTEx datasets. (A) Box plots illustrated the data distributions from TCGA and GTEx datasets before normalization. (B) PCA plot illustrated the cluster of the samples from TCGA and GTEx datasets before batch effect removal. (C) Box plots illustrated the data distributions from TCGA and GTEx datasets after normalization. (D) PCA plot illustrated the cluster of the samples from TCGA and GTEx datasets after batch effect removal. [file Image_2.PDF]

**A**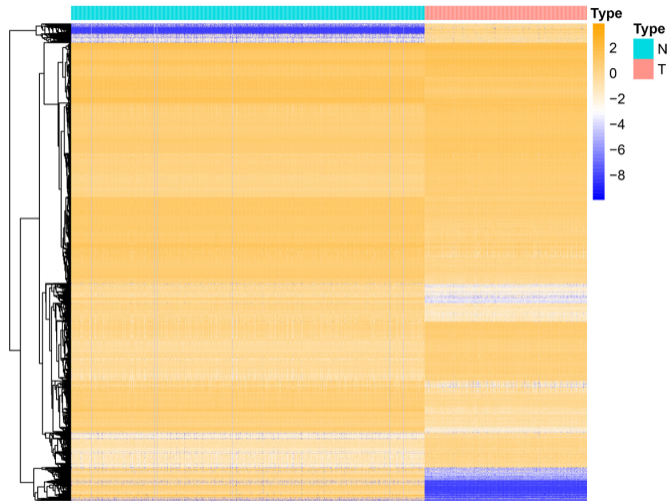**B**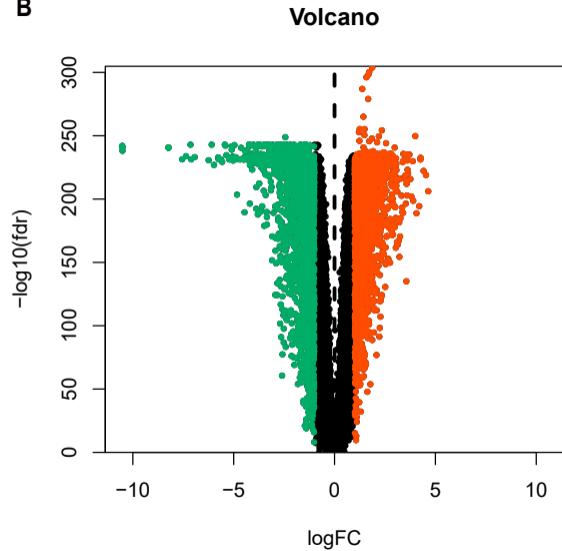

Supplement: FIGURE S3 — (A) Heatmaps showing that the 5,490 differentially expressed genes (DEGs) can effectively distinguish tumors from non-tumor tissues after integrated analysis. (B) Volcano plot presenting DEGs between LGG and non-tumor tissues. Red dots, and green dots represent up-regulated genes, and down-regulated genes, respectively. [file Image_3.PDF]

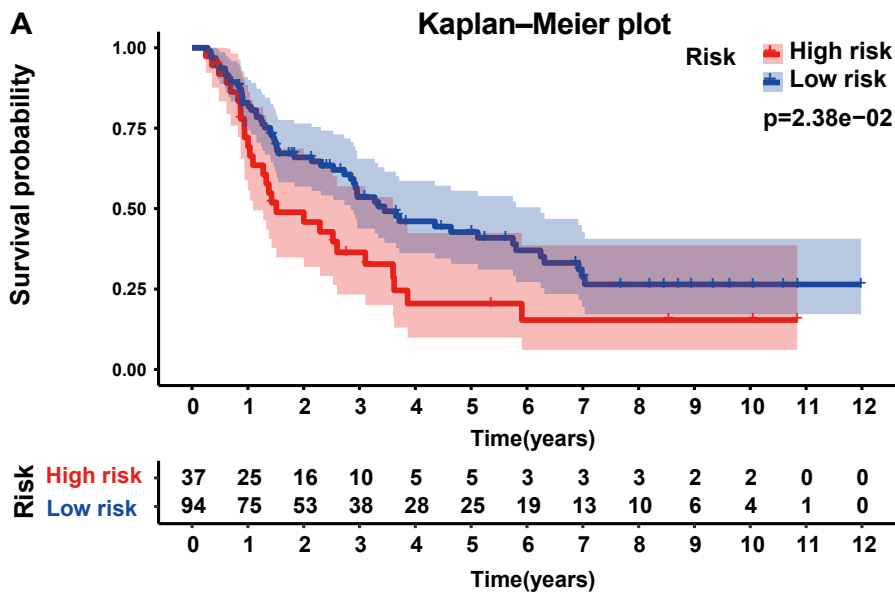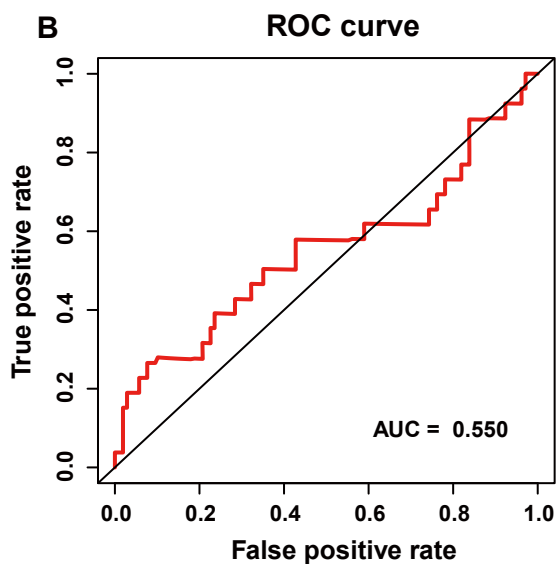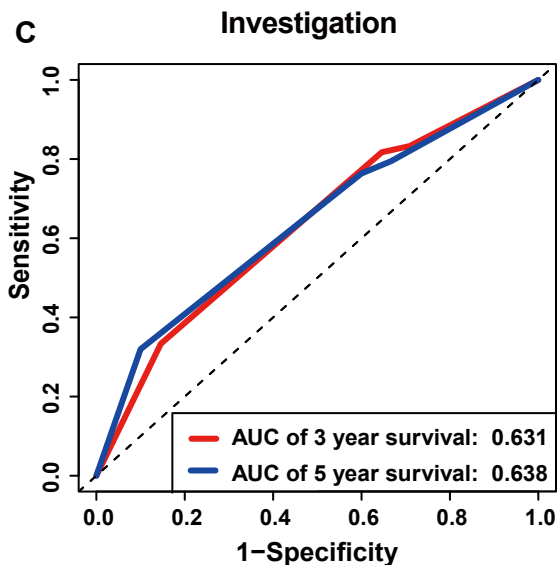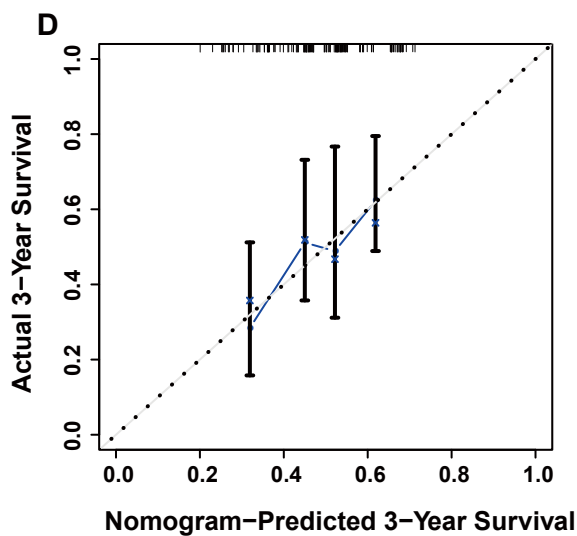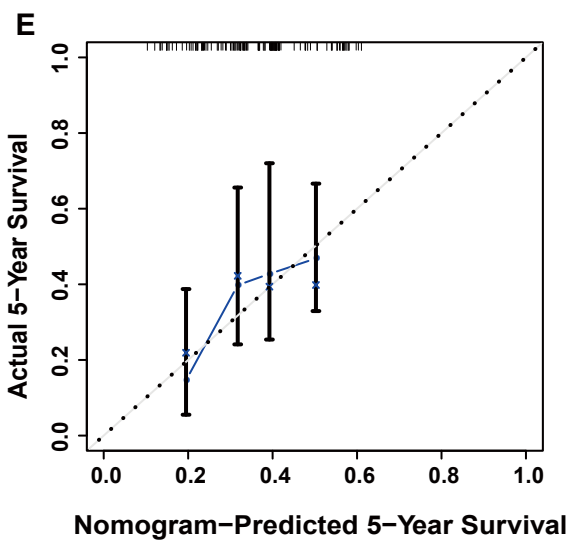

Supplement: FIGURE S5 — Investigating the application of six genes based signature in recurrent LGG. (A) Kaplan-Meier plot for overall survival based on risk score of the six gene based signature of recurrent LGG patients in CGGA cohort. (B) ROC curve based on the risk score for diagnostic competence verification of recurrent LGG patients in CGGA cohort. (C) Time-dependent ROC curve based on the six genes based risk score for 3-, and 5-year overall survival probability of recurrent LGG patients in CGGA cohort. Calibration curve for predicting probabilities of patients’ 3-year (D), and 5-year (E) overall survival of recurrent LGG patients in CGGA cohort. [file Image_5.PDF]

**A**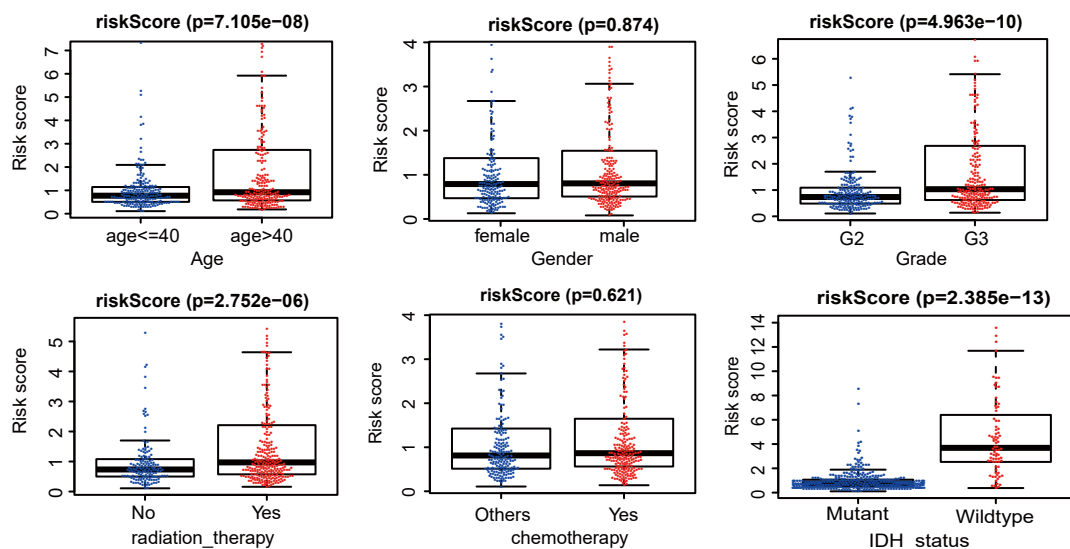**B**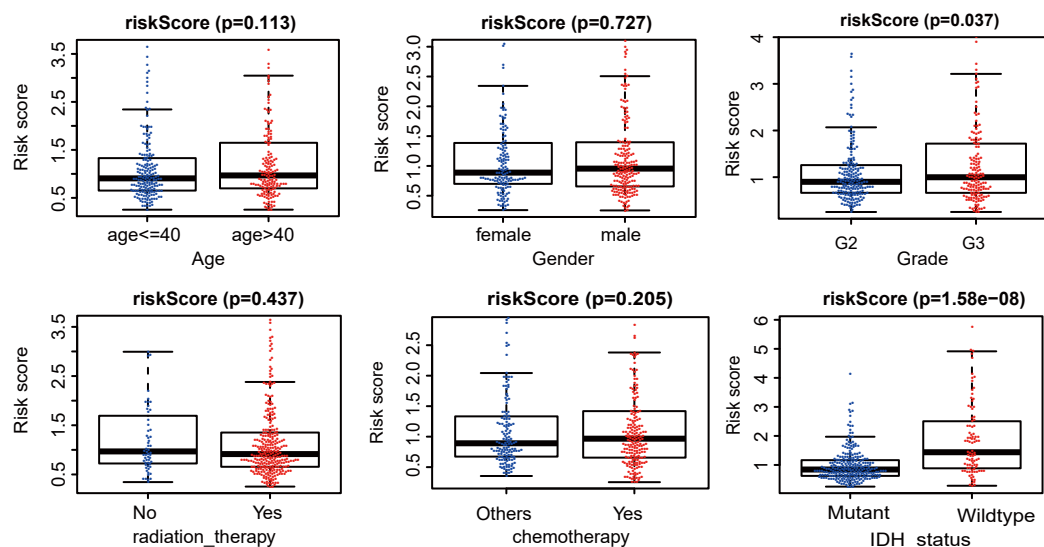**C**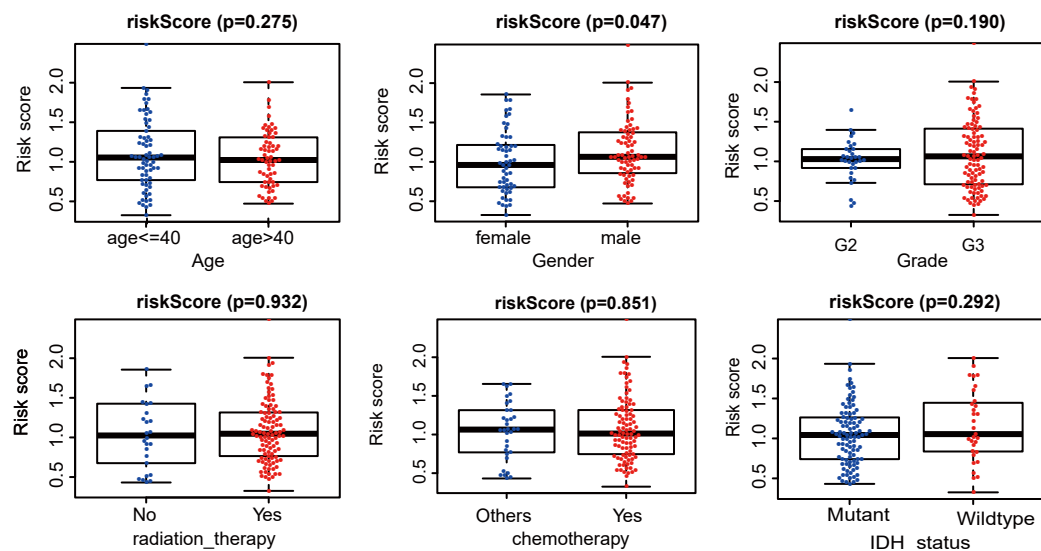

Supplement: FIGURE S6 — Association between risk score and clinical-pathological parameters. Association between risk score and age, gender, grade, radiotherapy, chemotherapy, and IDH mutation status of primary LGG patients in TCGA cohort (A), in CGGA cohort (B), while patients of recurrent LGG patients in CGGA cohort are shown in (C). [file Image_6.PDF]

**A**

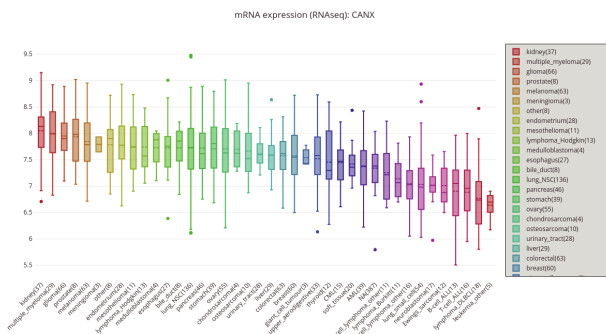

## B

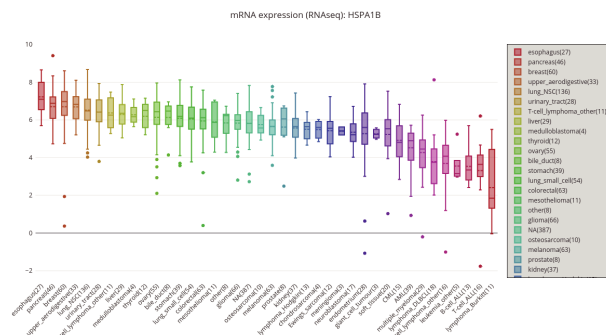

**C**

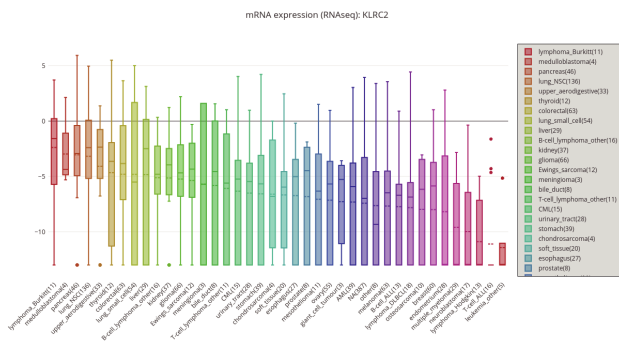

D

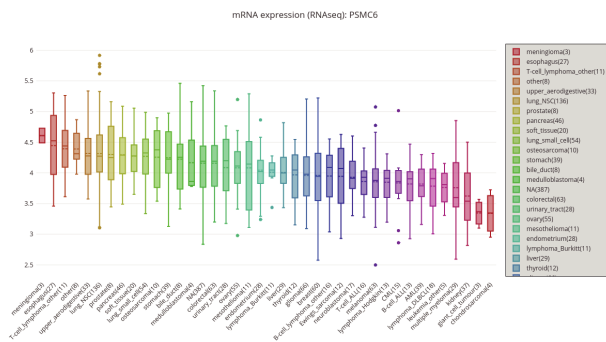

## E

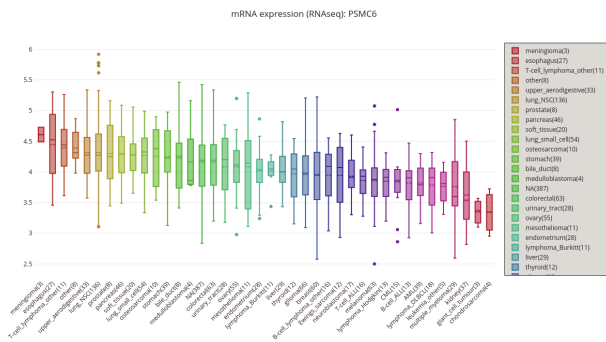**F**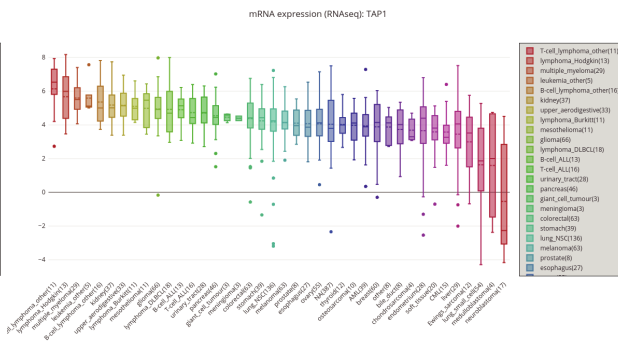

Supplement: FIGURE S8 — Expression data were sorted by the tumor type. The expression of the CANX (A), HSPA1B (B), KLRC2 (C), PSMC6 (D), RFXAP (E), and TAP1 (F) in Cancer Cell Line Encyclopedia. [file Image_8.PDF]

**A**

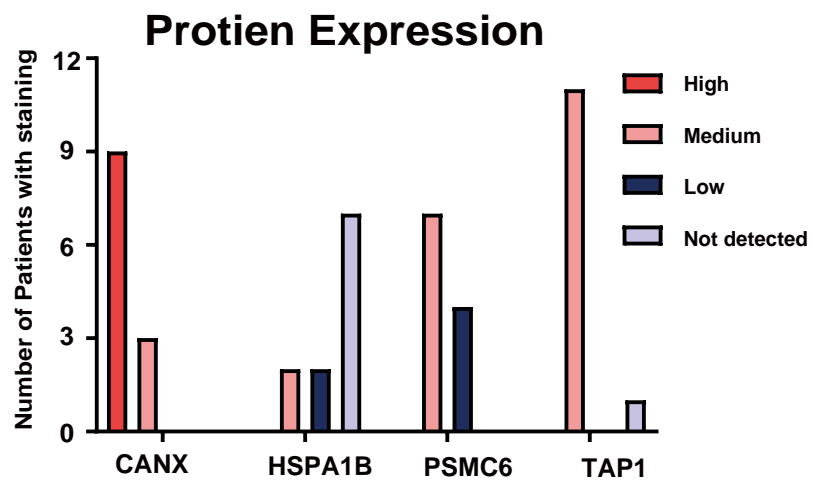

**B**

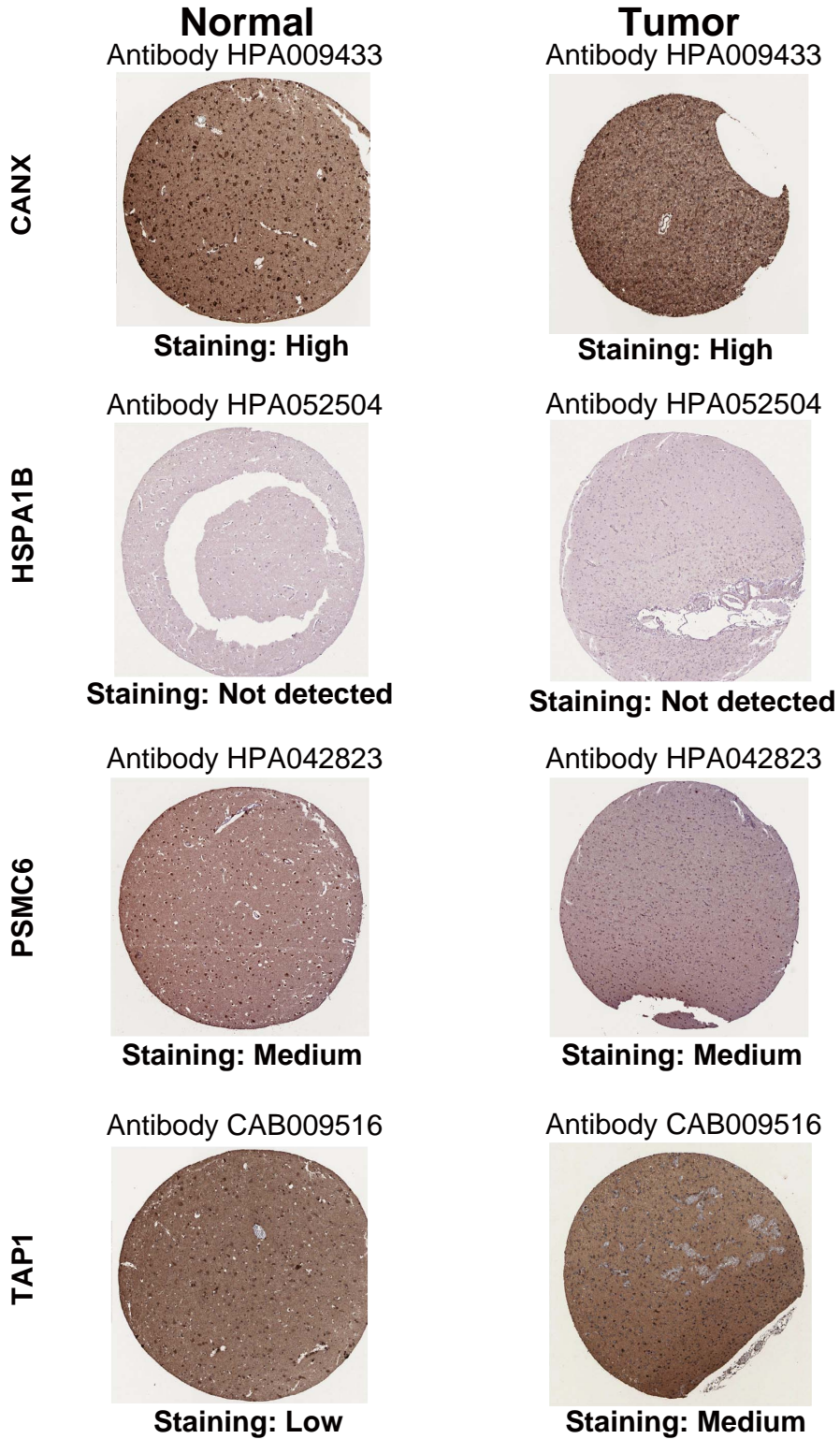

Supplement: FIGURE S9 — Number of patients with staining (A). The typical protein expression of six genes of immunohistochemistry (IHC) images in LGG tissue and paired non-tumor samples (B). Data was queried from the human protein atlas (https://www.proteinatlas.org/). [file Image_9.PDF]
