# Supplementary material for: Novel Immune-Related Gene Signature for Risk Stratification and Prognosis of Survival in Lower-Grade Glioma
Source: Front Genet. 2020 Apr 15;11:363. doi: 10.3389/fgene.2020.00363 (PMC7174786; doi:10.3389/fgene.2020.00363)
Supplement: FIGURE S4 — Schoenfeld residual plots showing P value of all factors were greater to 0.05. [file Image_4.PDF]

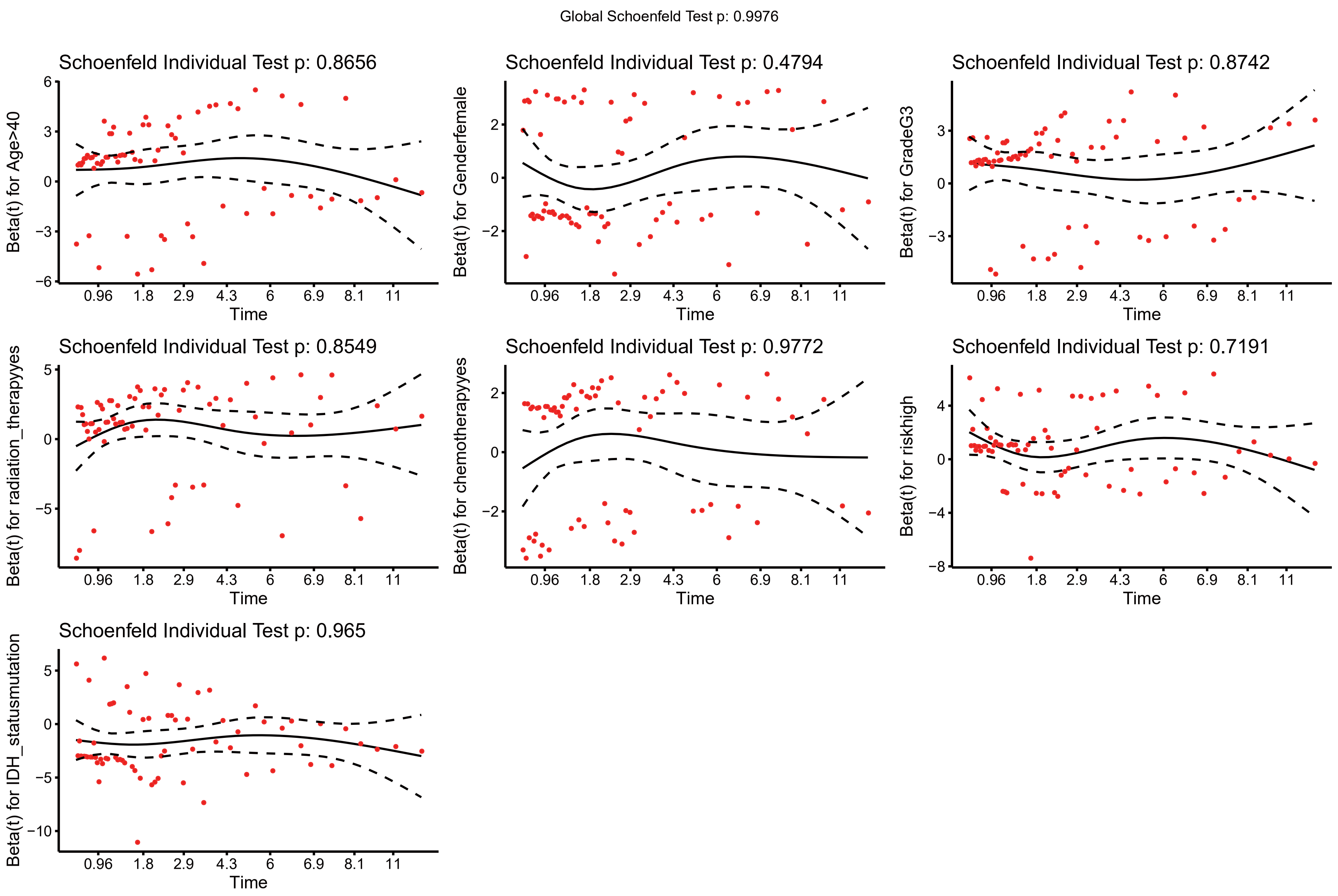

Global Schoenfeld Test p: 0.9976
Schoenfeld Individual Test p: 0.8656
Beta(t) for Age>40
Schoenfeld Individual Test p: 0.4794
Beta(t) for Genderfemale
Schoenfeld Individual Test p: 0.8742
Beta(t) for GradeG3
Schoenfeld Individual Test p: 0.8549
Beta(t) for radiation_therapyyes
Schoenfeld Individual Test p: 0.9772
Beta(t) for chemotherapyyes
Schoenfeld Individual Test p: 0.7191
Beta(t) for riskhigh
Schoenfeld Individual Test p: 0.965
Beta(t) for IDH_statusmutation
Time
0.96
1.8
2.9
4.3
6
6.9
8.1
11
