# Supplementary material for: Novel Immune-Related Gene Signature for Risk Stratification and Prognosis of Survival in Lower-Grade Glioma
Source: Front Genet. 2020 Apr 15;11:363. doi: 10.3389/fgene.2020.00363 (PMC7174786; doi:10.3389/fgene.2020.00363)
Supplement: FIGURE S7 — The differential expressed T cell associated activated and inhibitory genes, macrophage chemo-attractant and phagocytosis related genes between high and low risk groups in primary LGG. [file Image_7.PDF]

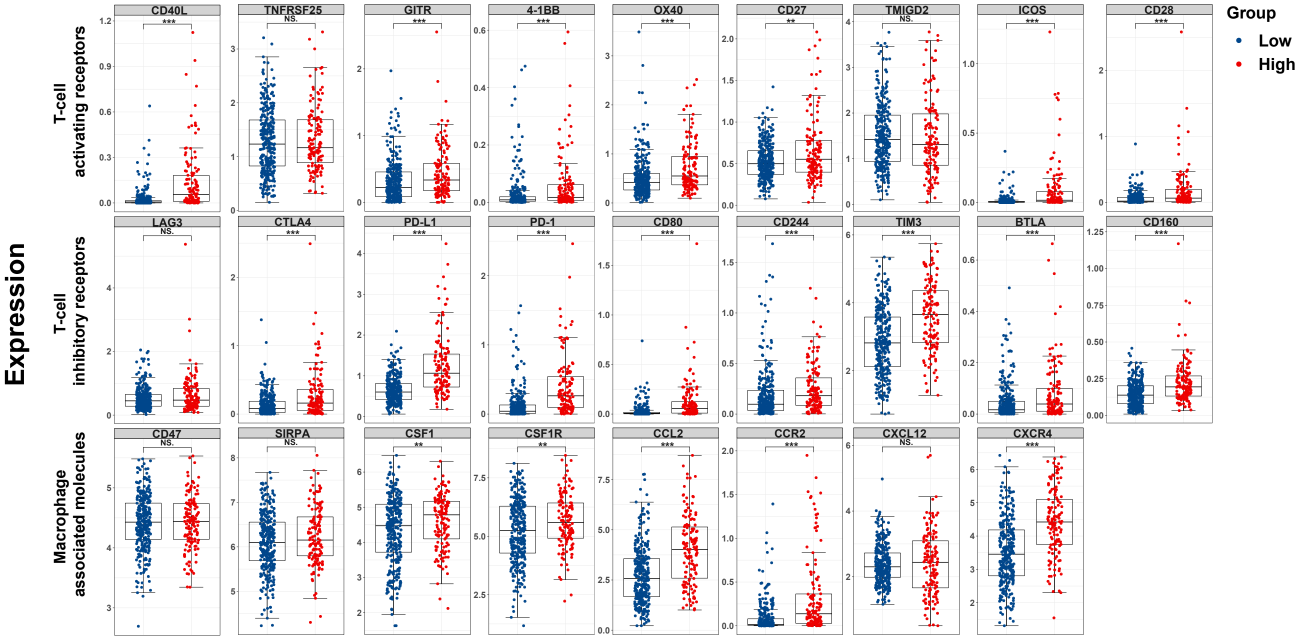

Expression
T-cell activating receptors
T-cell inhibitory receptors
Macrophage associated molecules
Group
Low
High
CD40L ***
TNFRSF25 NS.
GITR ***
4-1BB ***
OX40 ***
CD27 **
TMIGD2 NS.
ICOS ***
CD28 ***
LAG3 NS.
CTLA4 ***
PD-L1 ***
PD-1 ***
CD80 ***
CD244 ***
TIM3 ***
BTLA ***
CD160 ***
CD47 NS.
SIRPA NS.
CSF1 **
CSF1R **
CCL2 ***
CCR2 ***
CXCL12 NS.
CXCR4 ***
